# 8.O. Workshop: New challenges for suicide prevention in the context of Covid-19

**DOI:** 10.1093/eurpub/ckac129.526

**Published:** 2022-10-25

**Authors:** Organised by: EUPHA-PMH, Chair persons: Johan Bilsen (EUPHA-PMH), Jutta Lindert (EUPHA-PMH)

## Abstract

The recent COVID-19 pandemic confronted many societies with profound public health challenges. Although primarily a ‘somatic’ health concern, it becomes clear that Covid-19 and its aftermath also proved to have a substantial impact on mental health and wellbeing of the population in general and on vulnerable subgroups, such as young people and the elderly, in particular. An important public mental health concern in this context is the possible adverse influence of this pandemic on suicidality. Recent studies show that during the outbreak of the coronavirus disease the suicide mortality did not rise, rather on the contrary. However, the impact of the increased number of people with mental distress related to Covid-19 on suicidal ideation as well as the long-term impact on suicide rates is unclear. Also the long-term impact of the Covid-19 restriction measures greatly hindering adequate mental healthcare services and suicide prevention initiatives is uncertain. This workshop will focus on suicidality trends since the pandemic onset in different countries, suicide figures, experiences in the field of suicide prevention and on attitudes towards seeking help. Ann John from UK will talk about suicide trends during Covid-19 pandemic and will present suicide data of 35 countries collected by the international Covid-19 Suicide Prevention Research Collaboration. Fabrice Jollant will present data about the impact of Covid-19 pandemic on suicide attempts in France, and will discuss in this context the new national suicide prevention strategy. John Cachia will show the importance of a broad mental public health approach to suicide prevention in Malta. And finally, Saska Roskar from Slovenia will show how prevention of suicidality in individuals working themselves in the field of mental health can be hindered by self-stigma.

**Key messages:**

• We must remain vigilant about the possible long-term mental health impact (including suicidal behavior) of the Covid-19 pandemic.

• Pandemics like Covid-19 highlight certain weaknesses in suicide prevention strategies.

